# New Insights in Molecular Mechanisms in Antimicrobial Resistance and Strategies in Anti-Biofilms

**DOI:** 10.3390/antibiotics12040634

**Published:** 2023-03-23

**Authors:** Junyan Liu, Zhenbo Xu, Yulong Tan, Ren-You Gan, Guanggang Qu, Dingqiang Chen

**Affiliations:** 1 College of Light Industry and Food Science, Guangdong Provincial Key Laboratory of Lingnan Specialty Food Science and Technology, Academy of Contemporary Agricultural Engineering Innovations, Zhongkai University of Agriculture and Engineering, Guangzhou 510225, China; 2 Department of Laboratory Medicine, The Second Affiliated Hospital of Shantou University Medical College, Shantou 515041, China; 3 Special Food Research Institute, Qingdao Agricultural University, Qingdao 266109, China; 4 Singapore Institute of Food and Biotechnology Innovation (SIFBI), Agency for Science, Technology and Research (A*STAR), 31 Biopolis Way, Singapore 138669, Singapore; 5 Shandong Binzhou Animal Science & Veterinary Medicine Academy, Binzhou National Economic and Technological Development Zone, Binzhou 256606, China; 6 Department of Laboratory Medicine, Zhujiang Hospital, Southern Medical University, Guangzhou 510280, China

This topical collection, entitled “Antimicrobial resistance and anti-biofilms”, was first launched in the journal *Antibiotics* in November of 2020. A total of six editors served as the guest editors, including Dr. Dingqiang Chen, Dr. Yulong Tan, Dr. Ren-You Gan, Dr. Guanggang Qu, Dr. Zhenbo Xu and Dr. Junyan Liu. In this topical collection, there are three major sub-topics, including “Antimicrobial resistance in microorganisms: epidemiology and molecular mechanism (led by Dr. Dingqiang Chen)”, “New antibiofilm strategy against fungal and/or bacterial biofilms (led by Dr. Yulong Tan)” and “Influence of Functional Material-Based Encapsulation/Delivery on the Antimicrobial and Antivirulent Effects of Natural Compounds (led by Dr. Renyou Gan)”. Currently, this topical collection has published 10 manuscripts, with a few manuscripts under review. After accepting the first manuscript in 2021, this topical collection has published 9 manuscripts since 2022.

As part of this topical collection, the first sub-topic was “antimicrobial resistance in microorganisms: epidemiology and molecular mechanism”. The emergence and worldwide spread of antimicrobial resistance in microorganisms is a significant threat to human health. The epidemiology of infection by resistant microbes is still not clear in many areas. Investigating the underlying mechanisms of antimicrobial resistance is important for the control of infections caused by resistant microorganisms. Studies in this research field have improved our understanding of the epidemiology and molecular mechanism of antimicrobial resistance. Four manuscripts regarding the prevalence of resistant microbes, the emergence and evolution of resistance, and the molecular investigation of resistance mechanisms have been published in this sub-topic. The first manuscript entitled “Antimicrobial Treatment on a Catheter-Related Bloodstream Infection (CRBSI) Case Due to Transition of a Multi-Drug-Resistant *Ralstonia mannitolilytica* from Commensal to Pathogen during Hospitalization” provides convincing evidence that *R. mannitolilytica* transitioned from a commensal to a pathogen in the respiratory tract of a patient during the hospitalization, leading to a CRBSI [1]. *R. mannitolilytica* has been widely accepted as a commensal and colonizer; thus, it is easily overlooked. By focusing on the distinctive clone of *R. mannitolilytica* that was first identified as a colonizer, but then as a causative agent after antimicrobial treatment, this manuscript has brought *R. mannitolilytica* to our attention and its relevant potential transition in clinical settings. The second manuscript entitled “Occurrence of *Serratia marcescens* Carrying blaIMP-26 and mcr-9 in Southern China: New Insights in the Evolution of Megaplasmid IMP-26” describes a multidrug-resistant *S. marcescens* strain that co-harbored *bla*_IMP-26_ and *mcr-9* on a megaplasmid pYL4.1 and also included a proposed evolutionary pathway of epidemic megaplasmids carrying *bla*_IMP-26_ [2]. The spread of multidrug-resistant enterobacteria strains poses a significant concern to public health, especially when the strain harbors metallo-beta-lactamase (MBL)-encoding and mobilized colistin resistance (mcr) genes, as these genetic components potentially mediate multidrug resistance. Whole genome sequencing and bioinformatics analysis, in combination with abundant databases, can be used as efficient tools to identify antimicrobial resistance elements and evolution pathways, aiding in our understanding of the behavior of clinical isolates. Alternatively, the prevalence of certain antimicrobial resistance microbes and determinants was worth investigating to understanding regional epidemiology. The third manuscript entitled “NDM Production as a Dominant Feature in Carbapenem-Resistant Enterobacteriaceae Isolates from a Tertiary Care Hospital” describes the prevalence of carbapenem-resistant Enterobacteriaceae (CRE) and carbapenemase production in a teaching hospital in Pakistan [3]. As a potential source of outbreaks in healthcare settings and associated with high rates of morbidity and mortality, 238 Enterobacteriaceae isolates were collected with phenotypic and genotypic features. It was concluded that the NDM is the main resistance mechanism against carbapenems and is dominant in this region. The last manuscript in this sub-topic, entitled “The 30-Day Economic Burden of Newly Diagnosed Complicated Urinary Tract Infections in Medicare Fee-for-Service Patients Who Resided in the Community”, describes a retrospective multicenter cohort study of adult beneficiaries in the Medicare fee-for-service (FFS) database with complicated urinary tract infections (cUTI) between 2017 and 2018 [4]. A total of 723,324 cases reported in Medicare beneficiaries who met the study criteria were included and the major driver of cUTI-related 30-day MS was concluded as acute care hospitalizations. It raised our concern and suggested that healthcare systems should develop well-defined criteria for hospital admissions that aim to avert hospitalizations in clinically stable patients and expedite the transition of patients to the outpatient setting to complete their care.

The second sub-topic was the “new antibiofilm strategy against fungal and/or bacterial biofilms”. Microorganisms in nature produce biofilms to resist the pressures of the environment. The outer layer of the mature biofilm is a polysaccharide protein complex secreted by bacteria, and the inner layer is the coated bacteria. Bacterial biofilm formation is a dynamic process, which is mainly divided in four stages—initial adhesion, irreversible adhesion, aggregation, and development and abscission. Biofilms can protect microbial cells from antibiotics, chlorine, detergents, host immune defense mechanisms, and other factors. The resistance of microbes to antimicrobial substances in biofilms is 10 times or even 1000 times higher than that of planktonic modes. At present, the conventional chemical or physical methods are unable to completely remove biofilms. Traditional antibiotics can kill the cells inside, but they will cause drug resistance. In addition, the “inactive” biofilm structure can still promote the adhesion and regeneration of the biofilms of other microorganisms. Therefore, in addition to the microbial cells in thin biofilms, the biofilm matrix itself should also be a target. As a result, novel antibiofilm strategies that are able to interfere with cells inside and disassemble the biofilm matrix are needed in order to inhibit biofilm formation and/or disrupt persistent biofilms. Three manuscripts have been published on this sub-topic. Bacteria can form biofilms in natural and clinical environments on both biotic and abiotic surfaces. A comprehensive understanding of bacterial biofilm formation and the regulation mechanisms may provide meaningful insights into antibiotic resistance due to bacterial biofilms. A review entitled “A Review of Biofilm Formation of *Staphylococcus aureus* and Its Regulation Mechanism” discusses an overview of *S. aureus* biofilms, including the formation process, structural and functional properties of the biofilm matrix, and the mechanism that regulates biofilm formation [5]. *S. aureus* is one of the most common pathogens of biofilm infections. The formation of biofilms can protect bacteria from being attacked by the host immune system and antibiotics; thus, bacteria can overcome external challenges. Therefore, clinical treatments for biofilm infections are currently encountering difficulties. To address this critical challenge, new and effective treatment methods need to be developed. Focusing on *S. aureus* biofilms, a manuscript entitled the “Eradication of *Staphylococcus aureus* Biofilm Infection by Persister Drug Combination” has demonstrated an important treatment principle, the Yin–Yang model, for persistent infections by targeting both growing and non-growing heterogeneous bacterial populations, utilizing persister drugs for the more effective eradication of persistent and biofilm infections [6]. The combination of drugs that demonstrated robust anti-persister activity, such as clinafloxacin or oritavancin, in combination with drugs that displayed high activity against growing bacteria, such as vancomycin or meropenem, was shown to completely eradicate *S. aureus* biofilm bacteria in vitro. This study had implications for the improved treatment of other persistent and biofilm infections in general. *Escherichia coli* is also a common biofilm former, which normally colonizes in the gastrointestinal tract of humans and animals a few hours after birth, and pathogenic *E. coli*, which is the most common cause of gastrointestinal infections, such as diarrheal disease caused by enterotoxigenic *E. coli* (ETEC), and extraintestinal infections, such as urinary tract infections (UTI) caused by uropathogenic *E. coli* (UPEC). UPEC has a propensity to build biofilms to resist host defense and antimicrobials. Recurrent UTI caused by multidrug-resistant, biofilm-forming *E. coli* is a significant public health problem. The manuscript entitled the “Evaluation of Catechin Synergistic and Antibacterial Efficacy on Biofilm Formation and *acrA* Gene Expression of Uropathogenic *E. coli* Clinical Isolates” has described the antibacterial, synergistic, and antibiofilm activities of catechin isolated from *Canarium patentinervium* Miq. against three *E. coli* ATCC reference strains and fifteen clinical isolates [7]. Catechin demonstrated significant bactericidal activity and strong synergy when combined with tetracycline at the MBC value. In addition, catechin substantially reduced *E. coli* biofilm by downregulating the *acrA* gene. In silico analysis revealed that catechin bound with a high affinity to AcrB proteins (PDB-ID: 5ENT), one of the key AcrAB-TolC efflux pump proteins, suggesting that catechin might inhibit the *acrA* gene indirectly by docking at the active site of AcrB protein.

The third sub-topic was the “influence of functional material-based encapsulation/delivery on the antimicrobial and antivirulent effects of natural compounds”. Many natural compounds exhibit antimicrobial and antivirulent effects. Different functional materials, such as nanoparticles (e.g., lipid-based, polymer-based, carbohydrate-based, protein-based, metal-based, and nucleic acid-based nanoparticles), hydrogels, as well as micro/nano emulsions and packaging materials, can be used to encapsulate/deliver natural compounds and further enhance their antimicrobial and antivirulent effects. Therefore, understanding the antimicrobial and antivirulent effects of these functional material-encapsulated/delivered natural compounds, as well as their underlying mechanism of action, can provide a scientific basis for their further applications as antibiotic alternatives. Studies on this sub-topic improve our understanding of the influence of functional material-based encapsulation/delivery on the antimicrobial and antivirulent effects of natural compounds. Three manuscripts regarding the production and characteristics of these functional material-encapsulated/delivered natural compounds, their in vitro and in vivo antimicrobial and antivirulent effects, and their potential applications, such as in food and medicine, have been published in this sub-topic. Targeting mono and bispecies biofilms, three types of natural products have been evaluated as novel antimicrobial and antibiofilm drugs. The first manuscript entitled the “Antibiofilm Effect of Cinnamaldehyde-Chitosan Nanoparticles against the Biofilm of *Staphylococcus aureus*” described the particle size, potential, morphology, encapsulation efficiency and in vitro release behavior of cinnamaldehyde–chitosan nanoparticles (CSNP-CAs) and their activity against *S. aureus* biofilms [8]. The synergy between CA and CSNPs demonstrates the combinatorial application of a composite as an efficient novel therapeutic agent against antibiofilm. It was suggested that CSNP-Cas should be applied in food preservation and other contexts, providing new ideas for food preservation. The second manuscript entitled the “Effect of Extracts, Fractions, and Isolated Molecules of *Casearia sylvestris* to Control *Streptococcus mutans* Cariogenic Biofilm” described the effects of extracts, fractions, and molecules (the ethyl acetate (AcOEt) and ethanolic (EtOH) fractions from Brasília (BRA/DF) and Presidente Venceslau/SP, Caseargrewiin F (CsF)) of *Casearia sylvestris* to control the cariogenic biofilm of *S. mutans* [9]. CsF and AcOEt_BRA/DF damaged *S. mutans* cells and influenced the expression of virulence genes. Thus, an effect against biofilms occurred after prolonged exposure due to the bacteriostatic and/or bactericidal capacity of a fraction and a molecule from *C. sylvestris*. Invasive plants efficiently colonize non-native territories, suggesting a great production of bioactive metabolites that could be effective antibiofilm weapons. Targeting bispecies biofilm formed by *S. aureus* and *C. albicans*, which are among the most studied microorganisms because of their frequency of isolation in the case of infections, the third manuscript entitled the “Valorization of Invasive Plant Extracts against the Bispecies Biofilm *Staphylococcus aureus*–*Candida albicans* by a Bioguided Molecular Networking Screening” described the antibiofilm activity of extracts from five invasive macrophytes (*Ludwigia peploides*, *Ludwigia grandiflora*, *Myriophyllum aquaticum*, *Lagarosiphon major* and *Egeria densa*) [10]. The aerial stem extract of *L. grandiflora* showed the highest antibiofilm activity. The biological, chemical and bioactivity-based molecular networking investigations of its fractions highlighted nine ions that correlated with the antibiofilm activity. The most correlated compound, identified as betulinic acid, inhibited bispecies biofilms regardless of the three tested strains, confirming its antibiofilm potential.

As concluded, the above manuscripts published in this topical collection provided comprehensive knowledge and insight, which contributes to our understanding of antimicrobial resistance epidemiology and molecular mechanisms, new antibiofilm strategies, and novel natural compounds for biofilm eradication, covering food and clinical fields. It may aid in further therapy and control of related pathogens.
